# Toward a transdiagnostic modifier for clinically significant loneliness in psychiatry: a conceptual mini review

**DOI:** 10.3389/fpsyt.2026.1863015

**Published:** 2026-08-18

**Authors:** Alison Warren

**Affiliations:** 1 Department of Clinical Research and Leadership, George Washington University School of Medicine and Health Sciences, Washington, DC, United States; 2 Department of Continuing Education, Harvard University Extension School, Cambridge, MA, United States

**Keywords:** implementation science, knowledge translation, loneliness, psychiatry, social determinants of health, social isolation, transdiagnostic

## Abstract

Loneliness, the feeling that arises from the subjective perception of inadequate social connection, is increasingly recognized as a significant and modifiable determinant of health with effects spanning mental, cognitive, and physical domains. Despite a robust evidence base linking loneliness to adverse mental and physical health outcomes, it remains insufficiently integrated into psychiatric assessment and diagnostic frameworks, limiting its visibility in a patient’s clinical picture and treatment planning. This paper proposes reconceptualizing clinically significant loneliness as a transdiagnostic diagnostic modifier within psychiatric practice. Loneliness is positioned as a multisystem exposure operating across diagnostic categories through shared neurobiological and psychosocial pathways. From a translational science perspective, this reconceptualization offers a pragmatic strategy to bridge the gap between evidence and practice by embedding a clinically actionable construct into routine care. Importantly, assessing loneliness alongside psychiatric diagnoses enhances understanding of patients’ lived experiences, providing critical contextual insight into how symptoms are experienced, maintained, and treated. Incorporating loneliness as a modifier supports improved risk stratification, treatment planning, and care coordination while preserving the contextual nature of the experience. Integrating loneliness into psychiatric assessment represents a feasible and scalable step toward more comprehensive, person-centered care.

## Introduction

Humans are social beings by nature (1). When social connection is lacking in quantity and/or quality, emotional and psychological distress manifests as the feeling usually referred to as loneliness (2), also described by researchers as subjective social isolation (1) or perceived social isolation (3). This subjective perception of inadequate or unsatisfactory social connection is conceptually and clinically distinct from objective social isolation marked by a lack of contact with others, as it reflects a perceived discrepancy between desired and actual social relationships (4, 5). This distinction is critical, as objective social isolation may or may not be accompanied by loneliness or emotional distress, whereas the subjective experience of social disconnection (regardless of subjective or objective origin) demonstrates consistent and robust association with adverse physical and mental health outcomes (4, 6–8).

### The heterogeneity of loneliness

Although loneliness is frequently discussed as a single construct, decades of theoretical and empirical scholarship demonstrate that it is a heterogeneous phenomenon that varies in duration, relational context, developmental significance, and subjective meaning rather than representing a uniform psychological state (2, 9–12). For example, Young et al. (12) argued that loneliness is not a singular experience but encompasses qualitatively different forms arising from diverse interpersonal, developmental, and situational circumstances. Building on this foundation, Perlman and Peplau’s cognitive discrepancy model conceptualized loneliness as the distress that arises when an individual’s desired social relationships differ from those they perceive themselves to have, emphasizing that loneliness reflects the subjective appraisal of social relationships rather than their objective quantity (2, 10). Consequently, individuals with similar levels of reported loneliness may differ substantially in the underlying causes, phenomenology, persistence, and clinical implications of their experiences (4).

One particularly important distinction exists between transient and chronic loneliness (12). Transient loneliness is a common, short-term, and often adaptive emotional response to temporary disruptions in social relationships (13), such as bereavement, relocation, interpersonal conflict, or other normative life transitions. In many circumstances, this experience functions as an evolutionarily adaptive signal that motivates individuals to restore meaningful social connection (4, 6, 14). In contrast, chronic loneliness reflects persistent perceived social disconnection that may become self-reinforcing through maladaptive cognitive, behavioral, and physiological mechanisms, including heightened sensitivity to social threat, negative interpretive biases, social withdrawal, and dysregulated stress responses (4, 15). Consistent with these theoretical models, research consistently demonstrates that chronic loneliness differs from transient loneliness in both severity and clinical significance. Individuals experiencing persistent loneliness report substantially greater psychological distress, depressive symptoms, poorer physical health, greater chronic disease burden, lower physical activity, higher rates of maladaptive health behaviors, and increased healthcare utilization than those experiencing transient or no loneliness (4, 13, 16, 17). These distinctions suggest that persistence, rather than the mere presence of loneliness, may be particularly relevant for psychiatric assessment and clinical decision-making.

Loneliness also varies according to relational context. Individuals may experience loneliness in relation to peer relationships, romantic partnerships, family relationships, caregiving roles, workplace belonging, or broader community participation, each reflecting distinct unmet relational needs and social expectations (9, 11, 12). Loneliness also varies according to relational and developmental context. These contexts influence not only why loneliness occurs but also how it is experienced, expressed, and potentially addressed. Across the lifespan, loneliness may serve different psychological functions and carry different clinical implications. During adolescence, temporary experiences of loneliness may accompany normative developmental processes such as identity formation, increasing autonomy from parents, and changing peer relationships without necessarily indicating psychopathology (11). In adulthood, loneliness may emerge during transitions involving intimate relationships, parenthood, caregiving, occupational roles, or relocation, whereas in later life it often reflects bereavement, retirement, declining health, or shrinking social networks (11, 18). Similarly, loneliness associated with peer exclusion during adolescence differs conceptually and clinically from loneliness following bereavement in later life, caregiver-related loneliness, or persistent feelings of disconnection despite frequent social interaction (9, 11, 18, 19). These diverse relational experiences reflect distinct unmet social needs, developmental tasks, and interpersonal challenges, meaning that similar levels of loneliness may have markedly different antecedents, subjective meanings, and clinical implications depending on an individual’s developmental stage and relational context (9, 11, 12).

Equally important is distinguishing loneliness from other forms of solitary experience. Objective social isolation refers to the quantity or frequency of social contact, whereas loneliness reflects the subjective perception that existing relationships are insufficient or fail to satisfy relational needs (4, 5). Likewise, solitude and aloneness are not inherently negative experiences and may even serve as a homeostatic counterbalance to social interaction. The capacity to be alone has long been described as an important developmental achievement associated with emotional maturity, secure attachment, autonomy, and self-reflection (20). Voluntary solitude may facilitate creativity, emotional regulation, contemplation, identity formation, personal growth, empowerment, and agency (20–22), whereas loneliness is characterized by unwanted perceived social disconnection accompanied by psychological distress (22, 23). Individuals may therefore experience solitude without loneliness, loneliness despite being surrounded by others, or objective social isolation without subjective distress. These distinctions are clinically important because the relevance of loneliness lies not in time spent alone, but in the persistence, subjective meaning, developmental context, functional consequences, and associated distress of the experience. Furthermore, chronic loneliness is not uniformly associated with psychopathology. While persistent loneliness increases vulnerability to adverse psychological outcomes, some individuals demonstrate resilience, evidenced by adaptive coping and stable functioning despite enduring feelings of social disconnection (24). This variability in how loneliness is appraised, experienced, and responded to emphasizes its inherent heterogeneity and cautions against assuming that all persistent loneliness is intrinsically pathological.

Recognizing the heterogeneity of loneliness is fundamental to the present proposal. The intention is neither to conceptualize all experiences of loneliness or aloneness as indicators of psychopathology nor to medicalize a universal human experience. Rather, this framework focuses on persistent, distressing, and functionally impairing loneliness that meaningfully influences psychiatric presentation, treatment engagement, prognosis, and recovery. Loneliness is not a unitary clinical phenomenon but a multidimensional experience whose significance depends on developmental stage, relational context, persistence, subjective appraisal, and functional consequences. These characteristics make loneliness poorly suited to conceptualization as a discrete psychiatric symptom or diagnosis. Instead, they support its consideration as a transdiagnostic modifier, that represents a clinically meaningful contextual factor that can inform diagnostic formulation, treatment planning, and prognostic assessment across diagnostic categories while preserving the complexity and diversity of the underlying experience.

### Loneliness experience across the lifespan

Importantly, loneliness should not be conceptualized as a uniform construct across the lifespan, as its lived experience is shaped by developmental stage as well as substantial heterogeneity in individual characteristics, social contexts, and life circumstances. Developmental and lifespan research demonstrates significant variation in the phenomenology, drivers, and consequences of loneliness across age groups (9, 11). In adolescence and young adulthood, one of the major peaks in prevalence, loneliness is often closely tied to peer belonging, identity formation, and social comparison processes (11, 25, 26). In emerging and full adulthood, loneliness may be shaped by broader relational, occupational, and socioeconomic factors (11, 27), while in older adulthood it is more frequently associated with bereavement, caregiving transitions, functional decline, reduced mobility, and shrinking social networks (9, 25, 28–31). Additionally, the complex experiences of being an informal caregiver regardless of age add a further layer of complexity to the experience of loneliness (32). A recent qualitative systematic review further supports this lifespan framing, finding that loneliness is experienced differently across life stages and shaped by developmental, psychological, and contextual factors (9). These developmental differences have important implications for psychiatric assessment and intervention, suggesting that a single screening threshold or standardized intervention pathway may not be equally appropriate across populations or developmental stages.

Negative health outcomes traverse these developmental stages. A substantial and converging corpus of evidence demonstrates that loneliness is associated with increased risk of depression and anxiety (16, 33), suicidality (34, 35), cardiovascular disease (36), cognitive decline (37), and premature mortality (38, 39). Meta-analytic findings further indicate that the magnitude of these associations is comparable to established behavioral risk factors such as smoking, obesity, and physical inactivity (38, 39).

In recognition of these findings, the U.S. Surgeon General (39) and World Health Organization (40) have identified loneliness and social isolation as a major public health concern requiring coordinated responses across healthcare and community systems (39). Despite this growing recognition and association with psychiatric disorders, loneliness remains insufficiently integrated into psychiatric diagnostic frameworks, limiting its routine assessment and its role in clinical decision-making (5, 41). Further, when routine assessment is feasible, effective assessment must also be accompanied by evidence-informed intervention pathways to ensure that identification of loneliness translates into meaningful clinical support.

### Psychiatric classification

Current psychiatric classification systems, including DSM-5-TR (42), have advanced diagnostic reliability and communication but remain largely categorical in structure, emphasizing symptom clusters and thresholds that may obscure cross-cutting determinants influencing illness trajectory and treatment response (43). Although emerging dimensional frameworks such as the Research Domain Criteria and the Hierarchical Taxonomy of Psychopathology highlight transdiagnostic processes, including social functioning, these models have not been fully translated into routine clinical care (43, 44). Emerging efforts to integrate dimensional approaches into psychiatric classification systems highlight the limitations of strictly categorical diagnoses and support the value of incorporating cross-cutting constructs that capture clinically meaningful variation across disorders (45). This shift provides a strong conceptual foundation for operationalizing loneliness as a diagnostic modifier within existing frameworks.

### Loneliness as an opportunity for translation

From a translational science perspective, this disconnect reflects a broader challenge in psychiatry, more specifically, the gap between robust evidence identifying social determinants of health and their systematic integration into research and clinical workflows (46, 47). A key challenge in addressing social determinants of mental health is their sheer breadth, which can overwhelm clinicians (47). Loneliness represents a particularly compelling case, as it is both strongly evidence-based and conceptually clear, yet underutilized in practice. While many social determinants are clinically important (e.g., housing instability, food insecurity, adverse childhood experiences, discrimination, financial strain, transportation barriers), loneliness has garnered less attention in routine assessment due to similar barriers such as time constraints, overlap with existing psychiatric symptoms, lack of standardized tools, and unclear referral pathways (48). Yet, it is particularly well-suited for integration into psychiatric care due to its clear conceptual definition, validated measurement tools, and direct relevance to patient-reported experience, making it a pragmatic and scalable entry point for operationalizing social determinants in clinical practice.

Despite the absence of standardized clinical guidelines for assessing and addressing such factors, even among patients already engaged in care, practical, scalable approaches exist to integrate key social determinants into psychiatric practice (47). This paper argues that conceptualizing clinically significant loneliness as a transdiagnostic modifier offers a pragmatic and scalable pathway to improve knowledge translation, enhance implementation, and deepen clinical understanding; the rationale for this reconceptualization, and its clinical and health system implications, are developed in the Discussion. At the same time, conceptualizing clinically significant loneliness within psychiatric assessment frameworks requires careful ethical and conceptual consideration. Loneliness is a universal yet profoundly individualized human experience shaped by relational, cultural, and social contexts, and there is legitimate concern that excessive clinical framing could contribute to the overpathologization or medicalization of ordinary social suffering (49, 50). At the same time, failing to recognize persistent and impairing loneliness within psychiatric assessment may obscure clinically meaningful contextual factors influencing symptom severity, treatment engagement, functional outcomes, and recovery trajectories. While loneliness has been conceptualized as a “debilitating psychological condition characterized by a deep sense of emptiness, worthlessness, lack of control, and personal threat” by seminal loneliness researchers (51, p. 2), a clinically consequential condition is conceptually distinct from a formal psychiatric disorder. The intention of proposing loneliness as a transdiagnostic modifier is therefore not to redefine loneliness as a psychiatric disorder or reduce it to an individual deficit, but rather to improve recognition of how perceived social disconnection may influence symptom severity, treatment engagement, recovery trajectories, and health outcomes across psychiatric conditions. In this sense, structured assessment may serve less as a mechanism of pathologization and more as a pragmatic strategy for contextualizing patient experience and facilitating appropriate support, referral, and care planning. Importantly, this proposal is intended to stimulate further interdisciplinary dialogue regarding how psychiatry can responsibly integrate social and relational determinants into clinical care without collapsing social suffering into disease categories.

### Defining the proposed modifier

For the purposes of this conceptual proposal, the term loneliness modifier does not refer to loneliness as a universal human experience or to all manifestations of perceived social disconnection. Rather, it refers specifically to clinically significant loneliness that is persistent, subjectively distressing, and sufficiently consequential to influence psychiatric presentation, treatment engagement, prognosis, functional impairment, or recovery. Importantly, the proposed modifier is not determined by loneliness severity alone. Its interpretation should incorporate the persistence of the experience alongside its developmental stage, relational context, subjective meaning, functional consequences, cultural context, and the individual’s motivation for solitude or social connection. Consequently, transient, developmentally normative, contextually appropriate, or voluntarily chosen experiences of solitude would not constitute a positive modifier in the absence of clinically meaningful distress or functional impairment.

The proposed modifier therefore reflects not loneliness itself, but the clinical significance of loneliness within an individual’s broader biopsychosocial context. As such, it is intended to function as a contextual element of psychiatric formulation rather than as a diagnosis, symptom, or binary indicator of loneliness. This approach aims to preserve the heterogeneity of loneliness while providing a structured framework for identifying forms of loneliness that are clinically relevant to psychiatric assessment, treatment planning, prognosis, and recovery.

## Methodology

This mini review is narrative and conceptual in nature and was designed to synthesize current evidence relevant to the proposed conceptualization of loneliness as a transdiagnostic modifier in psychiatry rather than to provide a systematic review or meta-analysis. The literature was identified through iterative searches of PubMed, PsycINFO, Scopus, HOLLIS, and Google Scholar using combinations of terms including loneliness, social isolation, psychiatry, mental disorders, social determinants of health, transdiagnostic, implementation science, and screening. Priority was given to recent systematic reviews, meta-analyses, seminal theoretical publications, clinical practice recommendations, and highly cited empirical studies relevant to psychiatric assessment, biological mechanisms, implementation, and measurement. Additional articles were identified through citation tracking of key publications.

### Aim of this review

The aim of this mini review is to examine the evidence supporting loneliness as a common denominator in psychopathology, discuss its potential value as a transdiagnostic modifier within psychiatric practice, and to evaluate its conceptual, clinical, biological, implementation, and ethical implications. Specifically, the review synthesizes current evidence regarding the role of loneliness across psychiatric disorders, discusses available assessment approaches, and proposes future directions for integrating loneliness into routine psychiatric assessment.

### Loneliness as a transdiagnostic determinant of mental health

Loneliness is highly prevalent across psychiatric populations and is consistently associated with adverse mental health outcomes (7, 52) and use of mental health services (53). Longitudinal studies demonstrate that loneliness predicts the onset and persistence of depression and anxiety, independent of baseline mental health status (16, 54). Loneliness has also been correlated with mood disorders, such as bipolar disorder, and can precede manic episodes (55). It has also been found to be significantly elevated during times of euthymia, and markedly prevalent in persons with bipolar disorder with psychotic features (56). In persons with severe mental illness, including schizophrenia spectrum disorders and major depressive disorder, loneliness may decrease quality of life and social functioning, worsen prognosis, and increase suicidality (57). Other severe psychiatric disorders such as eating disorders are also associated with disproportionate vulnerability to loneliness. Persons with concomitant eating disorders and loneliness have shown elevated psychopathology (general and specific to eating), suggesting that loneliness may be a relevant aspect of the clinical presentation (58). While there is less literature examining loneliness and posttraumatic stress disorder, available literature suggests an increase in loneliness in this patient population as well (3).

Each of these relationships is bidirectional, such that loneliness contributes to the development and maintenance of psychiatric symptoms while psychiatric conditions themselves may exacerbate social withdrawal and perceived isolation (4). This bidirectionality is central to understanding loneliness as a diagnostic modifier rather than as a discrete psychiatric symptom. Unlike symptoms that are embedded within a single disorder construct, loneliness operates as a cross-cutting contextual and experiential factor that may shape illness severity, treatment responsiveness, functional outcomes, and recovery trajectories across multiple psychiatric diagnoses simultaneously. In this sense, loneliness functions analogously to other clinically meaningful modifiers in medicine that influence prognosis and management without constituting the primary disorder itself, while potentially operating across multiple psychopathologies.

Importantly, recent meta-analytic evidence indicates that loneliness is not only common but pervasive among individuals with severe mental disorders. In fact, research indicates that individuals with serious mental illness experience loneliness at roughly twice the prevalence observed in the general population (59). A systematic review and meta-analysis found that approximately 59.1% of individuals with severe mental disorders experience loneliness, with similarly high rates of objective social isolation reported among individuals with schizophrenia spectrum disorders (52). These findings emphasize that loneliness is not merely a peripheral concern but a highly prevalent and clinically significant feature.

Beyond symptom presence, loneliness is associated with greater symptom severity, chronicity, and functional impairment (5, 60). Individuals experiencing loneliness are more likely to report persistent depressive symptoms and increased suicidality (33, 35). Evidence synthesized in The Lancet Psychiatry further demonstrates that loneliness is a significant predictor of new-onset mental health problems in the general population (54).

Importantly, loneliness extends beyond psychiatric diagnoses and functions as a shared risk factor across neurological and medical conditions, including dementia and cardiovascular disease (36, 37, 61). Taken together, these findings support conceptualizing clinically significant loneliness as a multisystem, transdiagnostic determinant that influences both the development and trajectory of mental and physical health conditions. From a translational standpoint, the consistency and strength of these findings suggest that loneliness represents a mature domain of evidence that is ready for clinical application. However, without a structured mechanism for integration into diagnostic and care processes, its potential to inform clinical practice remains underrealized.

### Biological and psychosocial mechanisms

The clinical relevance of loneliness is further supported by a well-established body of mechanistic research demonstrating its effects across multiple biological systems. Loneliness functions as a chronic psychosocial stressor that activates neuroendocrine, autonomic, and immune pathways implicated in both psychiatric and medical illness (6).

At the neuroendocrine level, loneliness has been associated with dysregulation of the hypothalamic–pituitary–adrenal axis, resulting in altered cortisol secretion patterns and impaired stress responsivity (4). It is also linked to dysregulated autonomic function, contributing to cardiovascular and metabolic dysregulation (36, 62). Inflammatory processes represent another key pathway. Loneliness has been associated with increased expression of pro-inflammatory genes and elevated circulating inflammatory markers, including cytokines implicated in depression, cardiovascular disease, and neurodegeneration (36, 63). These findings support the conceptualization of loneliness as a biologically embedded exposure with systemic effects.

Although loneliness is frequently discussed in relation to neurobiological stress pathways, cognitive and perceptual mechanisms are equally important to its persistence and clinical relevance. Cognitive models of loneliness suggest that perceived social disconnection is associated with hypervigilance to social threat, negative interpretive biases, expectations of rejection, and maladaptive social cognition that may reinforce withdrawal and perpetuate loneliness over time (15). These findings are clinically important because they help explain why interventions focused solely on increasing social contact often demonstrate limited effectiveness in the absence of approaches targeting cognition, relational expectations, and social perception. Furthermore, intervention evidence reflects this complexity. Meta-analytic findings suggest that loneliness interventions produce modest and heterogeneous effects overall, with interventions targeting maladaptive social cognition often demonstrating greater effectiveness than interventions focused solely on increasing opportunities for social contact (25, 64). These findings underscore the importance of avoiding overly simplistic assumptions that loneliness can be resolved through social referral alone and reinforce the need for multimodal, individualized, and developmentally responsive intervention approaches.

### Assessment and implementation considerations

Integrating loneliness into psychiatric assessment offers a meaningful opportunity to enhance both clinical understanding and care delivery. Given that more than half of individuals with severe mental disorders may experience loneliness, routine assessment has the potential to substantially improve identification of unmet social needs and inform more contextually appropriate interventions (52). Routine screening using validated measures can therefore facilitate identification of individuals experiencing perceived social disconnection and support more comprehensive evaluation (5).

### Loneliness assessment measures

A key advantage of conceptualizing clinically significant loneliness as a diagnostic modifier is the availability of several validated assessment tools that can be readily integrated into psychiatric workflows. The UCLA Loneliness Scale (UCLA-LS), originally 20 items, is among the most widely used measures of subjective loneliness in both clinical and epidemiologic research (65–67). For routine clinical practice, the three-item UCLA Loneliness Scale (UCLA-LS3) offers a particularly feasible alternative due to its brevity, acceptable psychometric properties, and demonstrated validity in two large population-based studies of community-dwelling adults (Hughes et al., 2004). The UCLA-LS3 assesses three core dimensions of perceived social disconnection, namely, lack of companionship, feeling left out, and feeling isolated from others, with scores ranging from 3 to 9, where higher scores indicate greater perceived loneliness. In the original validation studies, the UCLA-LS3 demonstrated acceptable internal consistency (Cronbach’s α = 0.72 in both original validation samples), while the full UCLA-LS Version 3 has consistently demonstrated excellent reliability across diverse adult populations (Cronbach’s α = 0.89–0.94) (67, 68). Another widely validated instrument, the de Jong Gierveld Loneliness Scale, distinguishes between emotional and social loneliness, providing additional contextual information that may be particularly valuable in psychiatric assessment and treatment planning. Across the six- and eleven-item versions, the instrument has demonstrated good psychometric performance in community-dwelling and older adult populations, with internal consistency generally ranging from approximately 0.70 to 0.90 depending on the version, subscale, and study population (69, 70).

While numerous tools exist, two additional instruments warrant consideration in psychiatric and clinical settings. The Loneliness Automatic Thoughts Questionnaire (LATQ) (71, 72) is a nine-item measure assessing the frequency of loneliness-related automatic thoughts over the past week using a five-point response scale. Grounded in cognitive models of loneliness, the LATQ captures maladaptive cognitive patterns associated with perceived social disconnection, including self-criticism, perceived difference from others, helplessness, and ruminative social thinking. These cognitive dimensions are conceptually distinct from broader affective loneliness measures and may be particularly relevant for identifying patients whose loneliness is maintained by maladaptive social cognition (71, 72). Accordingly, the LATQ may have particular utility in cognitively oriented assessment and intervention contexts.

The ALONE scale (73) is a brief five-item clinician-administered measure developed and validated for use in healthcare and geriatric populations. The instrument demonstrated convergent validity with the UCLA Loneliness Scale, and preliminary validation studies suggested that scores of 3 or greater may indicate clinically meaningful loneliness in medical settings (73). Together, these instruments reflect a range of approaches differing in construct focus, brevity, administration method, and intended clinical context, as summarized in **Table 1**.

**Table 1:** Comparison of validated loneliness measures for use in psychiatric settings.

Measure	Full name	Length	Primary focus	Key features	Strengths	Limitations	Clinical utility in psychiatric settings	Key references
UCLA-LS (v3)	UCLA Loneliness Scale Version 3	20 items	Subjective loneliness and perceived social isolation	Comprehensive multidimensional assessment of perceived loneliness and social dissatisfaction	Extensive psychometric validation; strong reliability and construct validity; widely used across clinical and research settings	Longer administration time may limit feasibility in fast-paced clinical workflows	Useful for comprehensive psychiatric assessment, research settings, specialty clinics, and longitudinal evaluation of loneliness severity	Russell et al. (65, 66); Russell (67)
UCLA-LS3	Three-Item UCLA Loneliness Scale	3 items	Brief assessment of subjective loneliness	Measures lack of companionship, feeling left out, and feeling isolated from others	Brief, pragmatic, easy to integrate into routine screening; validated in large population-based studies; low administrative burden	Less nuanced than longer scales; limited dimensional detail regarding emotional and social loneliness subtypes	Well-suited for routine psychiatric intake, primary care screening, longitudinal monitoring, and implementation within clinical workflows	Hughes et al. (68)
De Jong Gierveld Scale	De Jong Gierveld Loneliness Scale	6 or 11 items	Emotional and social loneliness	Differentiates emotional loneliness from social loneliness	Captures multidimensional aspects of loneliness; validated internationally across age groups	Slightly longer administration time; less commonly used in U.S. psychiatric workflows	Useful when distinguishing emotional versus social loneliness may inform treatment planning and psychosocial assessment	De Jong-Gierveld & Kamphuls (69); De Jong Gierveld & van Tilburg (70)
LATQ	Loneliness Automatic Thoughts Questionnaire	9 items	Loneliness-related maladaptive cognitions and automatic thoughts	Assesses rumination, negative self-appraisal, perceived social inadequacy, rejection sensitivity, and maladaptive interpersonal cognitions	Captures cognitive mechanisms underlying chronic loneliness; useful for CBT conceptualization and intervention planning	Less extensively validated than UCLA measures; narrower focus on cognition rather than overall loneliness severity	Particularly useful in psychotherapy settings, CBT interventions, and research examining maladaptive social cognition	Besser et al. (71); Flett et al. (74)
ALONE Scale	ALONE Scale	5 items	Clinical loneliness risk screening	Brief clinician-oriented screening tool developed for healthcare and aging populations	Designed specifically for clinical use; rapid administration; feasible in medical and geriatric settings; clinically pragmatic	Newer measure with smaller validation literature; less extensively studied across psychiatric populations	Useful for rapid screening in geriatric psychiatry, integrated behavioral health, primary care, and routine healthcare assessment	Deol et al. (73)

UCLA-LS, UCLA Loneliness Scale; UCLA-LS3, Three-Item UCLA Loneliness Scale; LATQ, Loneliness Automatic Thoughts Questionnaire; CBT, Cognitive Behavioral Therapy. All measures are self-report unless otherwise noted. Score interpretation should be used alongside clinical judgment and may require developmentally stratified calibration across psychiatric populations.

From an implementation science perspective, frameworks such as the Consolidated Framework for Implementation Research (CFIR) may provide a useful structure for evaluating the feasibility, acceptability, sustainability, and contextual determinants associated with integrating loneliness screening into psychiatric workflows (75, 76). Applying implementation frameworks may help identify barriers related to clinician burden, workflow integration, referral infrastructure, stigma, community resource availability, and organizational readiness, all of which are likely to influence successful adoption across clinical settings. Particularly relevant CFIR domains may include intervention characteristics (e.g., adaptability and complexity of screening tools), inner setting factors such as implementation climate and workflow compatibility, outer setting factors including community referral capacity, and characteristics of individuals such as clinician knowledge, beliefs, and self-efficacy regarding loneliness assessment (76). These domains were selected because they represent the primary determinants likely to influence successful adoption of loneliness assessment in routine psychiatric care. Specifically, intervention characteristics address the feasibility and adaptability of screening instruments; the inner setting captures workflow integration and organizational readiness; the outer setting reflects the availability of referral resources and community supports; and characteristics of individuals encompass clinician knowledge, beliefs, and confidence in assessing and responding to loneliness. Together, these domains directly address the practical challenges most likely to determine whether loneliness assessment can be successfully implemented in real-world psychiatric settings.

Patient scores can inform risk stratification and guide clinical decision-making, including the initiation of targeted interventions such as social prescribing, psychotherapy, or community-based referrals (41, 77). Embedding such tools within electronic health records and clinical workflows enables systematic identification while minimizing burden on clinicians and supporting coordinated, team-based care (46, 78).

Beyond risk identification, assessing loneliness provides critical insight into patients’ lived experiences. Psychiatric diagnoses often capture symptom patterns without fully contextualizing the relational environments in which those symptoms occur. Incorporating loneliness into assessment may allow clinicians to better understand how social disconnection shapes symptom expression, maintenance, and recovery (4). This enhanced contextual understanding has direct implications for treatment planning. Knowledge that a patient is experiencing loneliness alongside a psychiatric disorder can inform the selection of interventions that address both symptoms and social context, including social prescribing and community-based approaches (77). However, the transition from screening to intervention should not be viewed as straightforward or universally effective. Although social prescribing is generally promising, intervention evidence remains mixed and effect sizes are generally modest. Meta-analytic findings suggest substantial heterogeneity across loneliness interventions, with approaches targeting maladaptive social cognition often demonstrating greater effectiveness than interventions focused exclusively on increasing opportunities for social interaction (64). More recent developmental reviews similarly conclude that intervention effectiveness varies considerably depending on age, intervention design, delivery context, and baseline loneliness severity (25). Thus, a need for cautious implementation is warranted, along with reinforcement that screening alone is insufficient in the absence of appropriate, individualized, and contextually responsive care pathways.

### Ethical and equity considerations

The integration of loneliness into psychiatric practice raises important ethical considerations. Screening for loneliness without the capacity to provide meaningful interventions may create unmet expectations for patients and increase clinician burden (78). As emphasized in JAMA, social risk screening must be accompanied by appropriate resources and referral pathways to avoid unintended harm (78). As such, equity considerations are central, as loneliness is shaped by structural determinants including socioeconomic disadvantage, social exclusion, and environmental factors that intersect with disparities in mental and physical health outcomes (5). Addressing loneliness within psychiatric care therefore requires attention to broader systemic and community-level interventions.

Further, although the psychological suffering and associated psychosocial and physical health consequences of loneliness are well-established (8), conceptualizing it as a diagnostic modifier raises important questions about the potential for overpathologizing a universal human experience (79). Loneliness is a normative and evolutionarily adaptive signal that can motivate social reconnection, and its presence alone does not necessarily indicate pathology (4, 6). Recent philosophical and phenomenological work has further emphasized that chronic loneliness may reflect disruptions not only in social contact, but in shared meaning-making, reciprocity, and participation in social worlds, suggesting that loneliness cannot be fully reduced to an individual symptom or intrapsychic deficit (80). Accordingly, its clinical integration must be approached with nuance, ensuring that assessment distinguishes between transient, contextually appropriate experiences and persistent, impairing states that warrant intervention (5).

The clinical value of loneliness assessment ultimately depends on the availability of appropriate interventions and referral pathways, as identifying loneliness without the capacity to respond offers limited benefit and may create unmet patient expectations. Although loneliness interventions continue to evolve, evidence supports a range of approaches, including cognitive-behavioral interventions targeting maladaptive social cognition, which have demonstrated some of the strongest effects on reducing loneliness (25, 64); structured group interventions designed to foster meaningful social engagement (25, 64); peer support approaches that promote shared lived experience and social connectedness (81); social prescribing, which links individuals to community resources and social activities (77); and broader community-based programs that strengthen social participation and connectedness (5, 40). Importantly, intervention effectiveness varies considerably across populations and settings, reinforcing that loneliness screening should be implemented alongside individualized assessment and appropriate referral pathways rather than as an isolated clinical activity (25, 64).

## Discussion

Loneliness is a modifiable, transdiagnostic determinant of health that remains underrecognized in psychiatric practice. As defined earlier, the proposed modifier reflects the clinical significance of loneliness as determined by its persistence, associated distress, functional consequences, and developmental and relational context rather than the mere presence of loneliness itself. This section situates that evidence within a broader argument: that clinically meaningful loneliness is better conceptualized as a diagnostic modifier than a clinical symptom, and that this distinction carries concrete clinical health system value.

### Why a modifier, and not a symptom

A clinical symptom is, by definition, bound to a specific disorder construct: its diagnostic meaning is interpreted only in relation to the criteria of the disorder in which it is listed, and its documentation is typically confined to the chart of patients meeting that diagnosis (82). Loneliness does not behave this way. As reviewed above, it precedes, accompanies, and outlasts depressive and anxiety episodes (16, 54), is disproportionately prevalent in bipolar disorder, schizophrenia spectrum disorders, eating disorders, and, to a more limited extent, posttraumatic stress disorder (3, 55–58), and shapes symptom severity, chronicity, and functional impairment in a manner that is not specific to any single nosological category (5, 60). Its relationship to psychiatric illness is also bidirectional rather than criterion-like: loneliness contributes to the onset and maintenance of symptoms, while psychiatric illness itself drives withdrawal and perceived isolation (4). A construct with this profile cannot be adequately captured by attaching it as a symptom to one or two diagnoses; doing so would render it invisible in the majority of patients for whom it is nonetheless clinically relevant.

Conceptualizing clinically significant loneliness as a modifier, analogous to severity specifiers, course specifiers, and other contextual qualifiers already used across medicine and psychiatry, instead allows it to be assessed independently of, and layered onto, any primary diagnosis. This has several concrete advantages over a symptom-based approach. First, it preserves diagnostic specificity: primary diagnoses continue to be defined by their own criteria, while loneliness is recorded as an additional axis of clinical information rather than being folded into, or mistaken for, the disorder itself. Second, it supports consistency of assessment: because a modifier is assessed the same way regardless of the primary diagnosis, it enables systematic identification and cross-diagnostic comparison in a way that disorder-specific symptom criteria cannot. Third, it aligns with the direction of emerging dimensional and hierarchical models of psychopathology, which favor cross-cutting constructs over strictly categorical, disorder-bound criteria (43–45). Finally, framing loneliness as a modifier rather than a symptom helps guard against the overpathologization concern raised above: a modifier flags a contextual factor relevant to care, whereas embedding loneliness within diagnostic criteria risks recasting an ordinary, and at times adaptive, human experience as inherently pathological (6, 79, 80). In this vein, interpreting loneliness as a transdiagnostic modifier requires consideration of developmental context. Similar experiences of loneliness may reflect normative developmental transitions in one individual while signaling persistent relational distress or vulnerability in another, highlighting the importance of developmentally informed clinical formulation rather than uniform interpretation.

### Clinical value

Operationally, the proposed modifier is not intended to classify loneliness itself but to identify clinically significant loneliness that is relevant to psychiatric formulation. A positive modifier would therefore reflect persistent, distressing, and functionally meaningful perceived social disconnection rather than transient, developmentally normative, or voluntarily chosen experiences of solitude. With this in mind, loneliness could be incorporated into psychiatric assessment using either categorical or graded approaches informed by validated screening instruments such as those summarized in **Table 1**. Existing loneliness measures are not currently designed for diagnostic classification; however, brief validated tools may still provide clinically meaningful stratification for assessment and care planning purposes. Given the absence of universally accepted clinical thresholds, loneliness may currently be best conceptualized dimensionally rather than diagnostically, with scores interpreted alongside psychiatric symptoms, functional impairment, developmental stage, and psychosocial context. A graded framework (e.g., minimal, mild, moderate, or severe loneliness) may therefore offer greater clinical utility than a binary present/absent classification, although future validation studies are needed to establish clinically meaningful cutoffs across psychiatric populations and age groups.

Crucially, this framework enhances understanding of patients’ lived experiences. Psychiatric diagnoses, while essential, often provide limited insight into the relational and social contexts in which symptoms arise. Incorporating loneliness into assessment allows for a broader understanding of these contexts by capturing how perceived social disconnection shapes symptom expression, vulnerability, and recovery trajectories (5). For instance, individuals with similar depressive symptom profiles may experience markedly different illness courses depending on the presence or absence of loneliness; recognizing loneliness in this context provides clinicians with a more nuanced understanding of symptom persistence and treatment responsiveness (33). This added contextual layer can inform more tailored and effective treatment planning, including the integration of interventions that explicitly target social connection, such as interpersonal psychotherapy, group-based interventions, and community-based supports (77).

The high prevalence of loneliness among individuals with severe mental disorders, affecting an estimated majority of this population (52), further strengthens the case for its integration into routine psychiatric assessment. When a construct affects a majority of patients within high-risk clinical populations, its omission from diagnostic formulation represents a missed opportunity for improving care. Incorporating loneliness as a diagnostic modifier ensures that this highly prevalent and clinically meaningful dimension is systematically recognized, which in turn supports diagnostic depth and treatment relevance (52). In this way, loneliness functions not only as a risk factor but as a clinically meaningful driver that shapes both the lived experience and management of psychiatric illness.

### Health system and translational implications

From a translational science perspective, conceptualizing clinically significant loneliness as a diagnostic modifier offers a strategy for bridging the gap between evidence generation and clinical implementation. Loneliness has accumulated a mature body of epidemiologic, mechanistic, and clinical evidence, yet this knowledge remains largely absent from routine psychiatric assessment and decision-making. Incorporating standardized loneliness assessment offers a practical mechanism for translating this evidence into actionable clinical information: routine assessment converts decades of observational and mechanistic research into a standardized clinical process that enables clinicians to identify loneliness consistently, incorporate it into diagnostic formulation when appropriate, guide individualized treatment planning, facilitate appropriate referrals, and monitor changes over time. Rather than remaining an abstract epidemiologic risk factor, systematic assessment operationalizes loneliness as a clinically meaningful, and system-level, construct.

At the health system level, embedding a brief, validated modifier into electronic health records and existing intake workflows is a comparatively low-burden change relative to the scale of its potential benefit, particularly given that loneliness already possesses validated instruments, a clear conceptual definition, and direct relevance to patient-reported experience (46, 48). Implementation science frameworks such as CFIR offer a structured way to anticipate the workflow, training, and referral-capacity barriers that determine whether such integration succeeds in practice (76). Realizing this benefit, however, depends on pairing assessment with adequate referral infrastructure and community resources; screening without the capacity to respond risks becoming a performative exercise that increases clinician burden without improving care (78).

Collectively, reframing loneliness as a transdiagnostic modifier offers a clinically meaningful and system-feasible framework for integrating a well-established social determinant of mental health into routine psychiatric assessment, thereby strengthening person-centered diagnostic formulation and treatment planning. This approach supports more personalized, contextually informed care and aligns with emerging dimensional models of psychopathology.

## Limitations & future directions

This paper has several limitations. As a narrative conceptual review, it does not employ a systematic review methodology and therefore is not intended to provide an exhaustive synthesis of the literature. Rather, the goal is to integrate evidence from psychiatry, neuroscience, implementation science, and public health to advance a conceptual framework. In addition, the proposal remains theoretical and empirical validation will be required before loneliness can be operationalized as a diagnostic modifier. Future work should evaluate diagnostic utility, clinical effectiveness, implementation feasibility, and potential unintended consequences across diverse psychiatric populations.

Translational science perspectives suggest that embedding loneliness into clinical workflows and leveraging existing clinical relationships represent a critical step toward aligning scientific knowledge with real-world care (83, 84). As psychiatry continues to evolve, incorporating loneliness into diagnostic and treatment processes offers a meaningful opportunity to improve outcomes and advance the integration of social determinants into mental health care. Several next steps are necessary before loneliness could be responsibly operationalized as a psychiatric modifier. First, developmentally stratified concordance studies are needed to determine clinically meaningful thresholds across psychiatric populations and age groups. Second, feasibility pilots should evaluate clinician acceptability, workflow integration, screening burden, and referral capacity within real-world psychiatric settings. Third, longitudinal studies should examine whether systematic assessment of loneliness improves treatment engagement, symptom trajectories, patient-reported outcomes, or care coordination. Finally, future work should explore whether alternative approaches, such as structured psychosocial formulations, dimensional assessment frameworks, or enhanced social history protocols, might achieve similar clinical benefits while minimizing concerns regarding overmedicalization.

## Conclusion

Psychiatry is increasingly moving toward models of care that recognize mental illness as emerging from dynamic interactions among biological, psychological, and social factors rather than from symptoms alone. Within this evolving landscape, loneliness represents a clinically meaningful dimension of patient experience that has remained largely peripheral to routine psychiatric assessment despite its pervasive influence across diagnostic categories. Reconceptualizing persistent and clinically significant loneliness as a transdiagnostic modifier does not seek to create a new diagnosis or redefine a universal human experience as pathology. Rather, it offers a framework for systematically recognizing a contextual factor that may shape illness presentation, treatment engagement, prognosis, and recovery across diverse psychiatric conditions. As evidence continues to accumulate, the challenge is no longer whether loneliness is clinically relevant, but how best to integrate this knowledge into psychiatric practice in ways that are evidence-informed, ethically responsible, and responsive to individual and community contexts. If validated through future implementation and clinical effectiveness research, this approach has the potential to advance more contextually informed, person-centered psychiatric.
